# The role of early intervention in improving the level of activities and participation in youths after mild traumatic brain injury: a scoping review

**DOI:** 10.2217/cnc-2016-0030

**Published:** 2017-08-10

**Authors:** Caroline van Heugten, Irene Renaud, Christine Resch

**Affiliations:** 1 Department of Neuropsychology & Psychopharmacology, Faculty of Psychology & Neuroscience, Maastricht University, Maastricht, The Netherlands; 2 School for Mental Health & Neuroscience, Faculty of Health, Medicine & Life Sciences, Maastricht University Medical Center, Maastricht, The Netherlands; 3 Limburg Brain Injury Center, Maastricht, The Netherlands; 4 Revant Rehabilitation Center, Breda, The Netherlands

**Keywords:** activities, children, participation, traumatic brain injury

## Abstract

Mild traumatic brain injury in children can lead to persistent cognitive and physical symptoms which can have a negative impact on activities and participation in school and at play. Preventive treatment strategies are preferred because these symptoms are often not recognized and therefore not treated adequately. In this review clinical studies investigating interventions directed at pediatric mild traumatic brain injury are summarized, and clinical recommendations and directions for the future are provided. Results show that the literature is scarce and more high quality studies are needed. Information and education about the injury and its consequences are recommended, with additional follow-up consultation, including individualized advice and reassurance. The interventions should be family-centered and, ideally, the return to activity and participation should be graded and done step-by-step.

Accidents can happen. Children and adolescents are often involved in accidents leading to traumatic brain injuries (TBI). The incidence of TBI in children between 0 and 18 years is 280–1373 per 100,000, but there is a large variation between studies and countries; most of these injuries are mild (mTBI) [1–8]. Most children recover completely after an mTBI, but 6–43% of children experience postconcussive symptoms (PCS) up to 6 months after the injury and beyond [9–12]. Persistent symptoms are found in the areas of physical, cognitive, emotional and behavioral functioning [13–16]. These consequences can lead to limitations in activities and participation such as returning to school and play [17–20]. Pediatric mTBI can also affect health-related quality of life (HRQOL) [21]. Children with PCS had significantly lower HRQOL scores at 4, 8 and 12 weeks postinjury than children without PCS and normal controls. Children without PCS had lower HRQOL scores than the norms at 4 and 8 weeks postinjury. School functioning scores were lower at all time points, regardless of the presence of PCS.

In general, children suffering the more severe forms of TBI (i.e., moderate and severe) are followed and receive rehabilitation treatment, but children with mTBI do not [22]. Both for professionals and for parents, the cognitive, emotional and behavioral problems are difficult to recognize and are therefore underestimated, underdiagnosed and not treated adequately [23]. Delayed recognition may, however, lead to unnecessary chronic and disruptive problems in activities and participation [13,24,25].

Several intervention strategies can be considered. First, all children and their parents can be given information and education on the possible consequences of an mTBI in order to prevent long-term problems. Second, children at risk of long-term problems can be identified at an early stage and information and education can be directed specifically toward this group. Early recognition and interventions are essential for children at risk of long-term problems [26,27]. It is, however, difficult to identify those at risk because the prognostic factors are not yet fully known. Third, information provision can be combined with routine follow-up aimed at detecting possible consequences. If disabling consequences are found, referral for treatment can be arranged. Finally, patients or parents who report consequences themselves, for instance to their general practitioner, can be referred for treatment. However, often this does not occur as these consequences are not recognized, either by the patients and parents themselves or by professionals. Accordingly, strategies for preventing long-term problems have been suggested to be the best treatment option [28,29].

In this paper we will review the available literature on early interventions for improving the level of activities and participation in children and adolescents with mTBI. On the basis of this overview we will formulate recommendations for clinical practice and suggest directions for future research.

## Review of the literature

We did not perform a systematic review with a predefined search strategy because of the limited resources on this topic. Instead we performed a scoping review which uses a more broad research question: inclusion and exclusion criteria can be developed *post hoc*; study quality is not a priority; the review may or may not involve data extraction and offer a more qualitative than quantitative synthesis of evidence [30]. However, we did make some decisions concerning the inclusion of studies and we did extract data. To begin, we used the definition of mTBI as defined by the American Congress of Rehabilitation Medicine, that is: ‘a Glasgow Coma Scale (GCS) score of 13–15 and at least one of the following: loss of consciousness of no more than 30 min; Post Traumatic Amnesia no longer than 24 h; any alteration in mental state at the time of the injury; focal neurological deficit(s) that may or may not be transient’ [31]. We searched studies using the terms ‘mild brain injury’, ‘mild traumatic brain injury’, ‘mild head injury’ and ‘concussion’ in combination with ‘children’/‘childhood’, ‘youth’, ‘adolescents’/‘adolescence’, ‘pediatric’/‘paediatric’ and ‘interventions’, ‘activities’ and participation’.

We selected clinical studies in which an intervention for children with mTBI was evaluated in the domains of activities and participation according to the framework of the International Classification of Functioning (ICF, WHO). Activities can be activities of daily life such as self-care, school, sports, hobby and play. Participation refers to the involvement of the children in life situations such as in domestic, community, social and civic life. We also considered family functioning or parent–child interaction as outcome domains. We did not include studies measuring outcome solely in terms of functioning such as motor functioning or cognitive functioning. We also did not include studies on interventions aiming at biochemical and neurochemical changes such as oxidative stress, inflammation and the neurometabolic cascade because these are mostly experimental and involve animal models, and outcome is measured mostly on the level of physiological and neurological functioning. Since pharmacological interventions are not primarily directed at improving the level of activities and participation, we excluded medication studies as well. If, however, medication was part of a more comprehensive program we did include the study.

Second, since the literature is still rather scarce in this area, we also considered studies in which children with mTBI were part of larger studies on moderate and severe pediatric TBI. Furthermore, studies that did not measure the level of activities and participation, but nevertheless investigated interventions for children with mTBI that might also be suitable for preventing problems with or improving the level of activities and participation, were included. Last, we discuss some potentially effective interventions from the literature on adult brain injury.

In addition to research papers, we considered reviews on interventions for children with (m)TBI [32–38] and searched for relevant references in these reviews. We extracted only studies in which interventions were evaluated. Papers describing treatment programs without an outcome evaluation were not considered; if these papers contained relevant recommendations for future research or clinical practice these are taken into account in our discussion section. If the review considered adults and children, we selected only the studies investigating children. If multiple papers were published about the same study, these are discussed separately only when they concern a different sample.

## Summary of the evidence on (early) interventions in pediatric (m)TBI

The following paragraphs describe the various identified types of intervention (e.g., information and education, online family problem-solving (FPS) interventions, cognitive and physical rest), separating interventions that are primarily aimed at all children with (m)TBI from interventions that target specific complaints in a subgroup of children who experience negative symptoms and/or are at risk of experiencing them in the future.

Table 1 provides an overview of studies investigating the effectiveness of interventions for activities and participation of children with mTBI. It also shows studies where children with mTBI were part of a larger group of children with moderate and/or severe TBI. Table 1 is the main table in which conclusions are drawn. The appendix displays studies of interventions for children with mTBI that are not directed at activities and participation, but nevertheless might be suitable for achieving improvement in these domains. These studies and studies on adults with mild forms of brain injury are described in the text only as ‘additional information’.

**Table 1.** **Overview of studies into interventions for improving the level of activities and participation for children with mild traumatic brain injury.**

**Study and participants**		**Intervention**		**Outcomes**	**Ref.**

**Study (year), country**	**Study design, participants (n, injury, age)**	**Type**	**Start, duration and frequency**	**Measurement time points, measures and results^1,2^**	
Casey *et al*. (1987), USA	Prospective, randomized controlled trial n = 321 (intervention n = 153, control n = 168) Injury: mTBI Age: 6 months–14 years	Information and education/follow-up consultancy Content: discharge interview, take-home booklet, follow-up telephone call Control intervention: care as usual	Start: directly at discharge Duration/frequency: interview and phone-call once, consult booklet when needed	Time points: 1 month postinjury (by telephone, n = 204) Physical (e.g., general health, resistance) –No effect Social limitations in daily activities: role activity index –No effect Behavior (e.g., behavioral screening, mental health survey) –No effect	[39]

Ponsford *et al*. (2001), Australia	Nonrandomized, controlled study n = 119 (intervention n = 61, control n = 58) Injury: mTBI Age: 6–15 years	Information and education Content: neuropsychological assessment, symptom booklet, coping strategies Control intervention: care as usual	Start: within 1 week postinjury Duration/frequency: one-time early face-to-face assessment. Information booklet could be consulted whenever needed	Time points: 3 months postinjury Postconcussion symptoms (PCSC) +Less overall symptoms, less headaches, less irritability, better judgement Irritability, inattentiveness and conduct (Rowe BRI) +Fewer behavioral problems –No effects on restlessness and sleep Behavior (CBCL) +Less problems with somatic symptoms, anxiety, social, thought, attention, delinquency, aggression, total problems and internalizing –No effects on externalizing problems Behavior in daily activities (VABS) –No effect of the intervention Neuropsychological measures –No intervention effects	[40]

Narad *et al*. (2015); Wade *et al*. (2015)/(2014), USA	Randomized controlled trial n = 132 (intervention n = 65, control n = 67) Injury: complicated mild/moderate TBI (n = 40 in CAPS, n = 41 in IRC) and severe TBI All participants had been hospitalized overnight Age: 12–17 years	Online family problem-solving training Content: CAPS Control intervention: IRC	Start: 1–7 months after hospitalization for TBI (M = 3.54) Duration/frequency: CAPS: 6 months, 7–11 sessions IRC: ≥1 h per week	Time points: baseline, 6 months, 12 months, 18 months Parent-reported and teen-reported conflicts (PSDRS) +Decreased conflicts in the CAPS group at 6 months for adolescents with complicated mild/moderate TBI, but not after 6 months Effective communication/observed parent–teen interactions (IFIRS) –No change over time for adolescents with complicated mild/moderate TBI in the CAPS group Parent-rated problem-solving (FAD) +Improved at 6 months, independent of group or TBI severity Child and adolescent functioning (CAFAS) +Everyday functioning in school and the community improved more over time (18 months) for the CAPS group than for the IRC group –The CAPS had no effects on home functioning, behavior, mood or thinking Externalizing and internalizing adolescent behavior (CBCL) +Older adolescents in the CAPS group had less externalizing problems than adolescents in the IRC group. No group differences in effects on internalizing behaviors	[41–43]

Wade *et al*. (2005), USA	Quasi-experimental pretest/post-test design n = 6 Injury: Complicated mild/moderate (n = 2) and severe (n = 4) TBI Age: 5–16 years	Online family problem-solving training Content: online FPS	Start: >15 months postinjury (M = 18.67 months, SD = 4.93) Duration/frequency: eight core sessions and four supplementary sessions	Time points: baseline, postintervention Child social competence and antisocial behavior problems (HCSBS) +Reduced parent-reported antisocial behaviors –No effects on social competence Parent–child conflict (IBQ-C/P, PARQ) +Reduced adolescent-reported conflicts about school –No effects on global conflict ratings and parent-reported school conflict Family functioning (FAD) –No effects Executive function skills behavior (BRIEF-GE) –No effects	[44]

Wade *et al*. (2006), USA	Randomized controlled trial n = 32 (intervention n = 16, control n = 16) Injury: complicated mild/moderate (67.6%) and severe (32.4%) TBI Age: 5–16 years	Family problem-solving training Content: FPS Note. Participants in the FPS group additionally received care as usual (n = 16) Control intervention: care as usual = standard medical care (n = 16)	Start: until 18 months after injury (M = 8.78, SD = 4.53) Duration/frequency: seven core sessions, four supplementary sessions (M = 8.31 sessions completed, range: 3–11)	Time points: baseline, postintervention Child adjustment (CBCL) +Reduced behavioral problems at post-test for FPS group Parental psychological distress (BSI) – No effects Parent–child interaction (CBQ) –No effects +In addition, parents of the FPS group reported improvements in knowledge of problem-solving strategies and understanding their child better	[45]

Wade *et al*. (2006), USA	Randomized controlled trial n = 39 (intervention n = 19, control n = 20) Injury: complicated mild/moderate (65% in IRC and 75% in FPS group) and severe TBI Age: 5–16 years	Online family problem-solving training Content: adapted online FPS Control intervention: IRC	Start: between 1 and 24 months after injury (M = 13.73, SD=3.16) Duration/frequency: 14 self-guided sessions: 8 core session and 6 supplementary sessions	Time points: baseline, postintervention Family functioning (FAD problem-solving and communication subscales) –No effects Child adjustment (CBCL) –No effects Social competence and antisocial behavior (HCSBS) +Improvement in self-management for the FPS group –No improvement on total score or peer relations	[46]

Wade *et al*. (2008) USA	Quasiexperimental pretest/post-test design n = 9 Injury: complicated mild, moderate and severe TBI Age: 11–18 years	Online family problem-solving training TOPS Two conditions were compared: TOPS-audio (n = 5), where families could have the text on the website read out loud to them, and TOPS-no-audio (n = 4)	Start: 24 months after injury Duration/frequency: 10 self-guided core sessions and up to 4 out of 6 additional sessions	Time points: baseline, postintervention Child behavior problems (CBCL) +Less internalizing symptoms –No effects on externalizing symptoms or total score Daily executive functioning (BRIEF) –No effects Depression (CDI) +Lower levels of depression postintervention Parental distress (SCL-90-R) +Decline in parental depressive symptoms Parent-adolescent communication and conflict behavior (CBQ, Issues Checklist, Issues Severity Scales) +Reduced parent-reported conflicts –No change in intensity of conflicts	[47]

Wade *et al*. (2011), USA	Randomized controlled trial n = 41 Injury: complicated mild/moderate and severe TBI (GCS M = 9.58, SD = 4.56) Age: 11–18 years	Online family problem-solving training TOPS (see Wade *et al*., (2008)) (n = 20) Control intervention: IRC (n = 21)	Start: 8–10 months postinjury Duration/frequency: See Wade *et al*., (2008))	Time points: baseline, 8 months Child behavior problems (CBCL, YSR) + Participants with severe TBI in the TOPS group showed improved parent-reported internalizing symptoms – No effects for complicated mild/moderate TBI Parent-adolescent communication and conflict behavior (IBQ-C/P) + Decreased parent-reported conflict in TOPS group – No effect on adolescent reported parent-teen conflict after TOPS	[48]

Thomas *et al*. (2015), USA	Randomized controlled trial n = 88 (intervention n = 45, control n = 43) Injury: mTBI Age: 11–22 years	Cognitive and physical rest Intervention group: 5-day rest, then step-by-step return to activity Control group: 1–2 day rest, then return to school and step-by-step return to physical activity	Start: Within 24 h of mTBI Duration: 1–5 days	Time points: Day 0, day 1–3, day 3, day 4–10 and day 10 Balance (BESS) –No group differences Neurocognition (ImPACT, Ancillary Neuropsychiatric) –No group differences Postconcussive symptoms (PCSS) +More postconcussive symptoms during follow-up period in intervention group Physical and mental activity +The strict rest group reported less school and mental activity than controls. No group differences in physical activities	[31]

^†^Outcomes printed in bold are measures of activities and participation as categorized by the authors of the present review.

^‡^’+’ indicates a significant intervention effect. ‘-’ indicates no significant effect.

BESS: Balance error scoring system; BRI: Behavioural rating inventory; BRIEF: Behavioural rating inventory of executive functioning (GEC: General executive compound; MI: Metacognition index); BSI: Brief symptom inventory; CAFAS: Child and Adolescent Functional Assessment Scale; CAPS: Counsellor Assisted Problem Solving; CBCL: Child behavior checklist; CBQ: Conflict behavior questionnaire; CDI: Children's depression inventory; FAD: Family assessment device; FPS: Family Problem-Solving; h: Hour(s); HBI: Health behavior inventory; HCSBS: Home and Community Social Behavior Scale (HCSCBS-AB: Antisocial behavior; HCSBS-SC: Social competence); IBQ: Interaction behavior questionnaire (IBQ-C: Child-report; IBQ-P: Parent-report); IFIRS: Iowa Family Interaction Rating Scale; ImPACT: Immediate postconcussion assessment and cognitive testing; IQR: Interquartile range; IRC: Internet Resource Comparison; M: Mean; PARQ: Parent-adolescent relationship questionnaire; PCSC: Postconcussion symptoms checklist; PCSS: Postconcussion Symptoms Scale; PSDRS: Problem-Solving Discussion Rating Scale; Rowe BRI: Rowe behavioral inventory; SCL-90-R: Symptom Checklist-90 – Revised; SD: Standard deviation; TOPS: Teen Online Problem Solving; VABS: Vineland Adaptive Behavior Scales; YSR: Youth self-report.

It has to be noted that the definitions of TBI severity (i.e., mild, complicated mild, moderate or severe) were not consistent over the studies. Furthermore, the terms ‘mild brain injury’, ‘mild traumatic brain injury’, ‘mild head injury’ and ‘concussion’ may be used interchangeably [49,50]. The general clinical medical literature now uses mTBI [51]. The definitions used by the studies in this review vary. One study used the definition of the American Congress of Rehabilitation Medicine [40]. One study [31] made use of the Acute Concussion Evaluation form [52]. In two studies [53,54] the International Consensus on Concussion in Sport [55,56] was used. Other studies defined mTBI based on GCS scores (i.e., >12) and/or duration of loss of consciousness, duration of post-traumatic amnesia and presence/absence of focal neurological deficits [41–48,57,58]. For three studies [39,59,60], the definition of mTBI was described as, that is, ‘minor head injury’, or ‘diagnosed by a sports or rehabilitation medicine specialist’. Complicated mTBI was defined as a GCS score of greater than 12 with evidence of significant findings on clinical imaging [41–48,57]. Moderate TBI was defined as a GCS score of 9–12 [41–48]. Some studies combined complicated mTBI and moderate TBI and defined this group as moderate [41–48]. Severe TBI was defined as a GCS score of <9 [41–48].

### Information & education

Information about mTBI and education on signs and symptoms can be provided with the intention of improving the outcome of patients or their caregivers or both. Casey *et al*. were the first to study the effectiveness of an information and education protocol after childhood mTBI in reducing physical, social and/or behavioral problems postinjury [39]. Their intervention, consisting of a discharge interview during which the nurse explained a take-home booklet of symptoms that could be expected, instructions to follow at discharge and a follow-up telephone call 24 h after discharge, was found to be no more effective than the routine discharge sheet (i.e., a list of symptoms requiring reassessment at the hospital). However, in general, reporting of symptoms 1 month postinjury was low. A closer look at the data seemed to indicate that most symptoms at the 1-month follow-up occurred in children who had anxious parents, although this finding did not reach significance. Based on these findings, Casey *et al*. emphasize the importance of reassurance and education for parents about the signs and symptoms of minor head trauma (i.e., emphasizing that the symptoms are common and that they can be dealt with) [39]. This might aid children in returning to their daily activities and routines. Ponsford *et al*. developed just such an early education and reassurance intervention for children post-mTBI [40]. This study was the first to provide evidence that children who received a booklet describing symptoms and coping strategies within 1 week postinjury reported fewer PCS at 3 months postinjury, in comparison with those who did not receive this information. The intervention, however, had no direct effect on behavior in daily activities. However, the amount of difficulties that the study sample experienced in daily behavior before the interventions was already low. This low rate of symptoms might explain the lack of effect of the intervention in improving the functioning of children with mTBI.

Taken together, these studies seem to indicate that information and education interventions are useful in decreasing PCS in children with mTBI. These types of interventions could also be used to improve the level activities and participation of children with mTBI who report a decrease in or are at risk for problems in activities and participation (e.g., by preventing unnecessary absenteeism from school), but more research is necessary.

### Problem-solving interventions for families

Four different but very similar interventions, two offline and two online, were identified in the literature for improving family and adolescent problem-solving skills following childhood TBI. The Counsellor Assisted Problem Solving (CAPS) intervention, the FPS intervention, the online FPS intervention, and the Teen Online Problem Solving (TOPS) intervention all provide therapist-guided problem-solving training to adolescents with TBI and their families. In six to eight core sessions and, depending on the families’ needs, up to four additional sessions, self-guided online learning of problem-solving skills, communication, self-management and self-regulation, as well as video-counseling with a therapist are offered. The (non-online) FPS intervention differs slightly, since the therapist and the families met at the families’ homes or at the clinic for the therapy sessions, instead of participating in video-counseling. In most of the studies in the CAPS, (online) FPS or TOPS intervention, Internet Resource Comparison was used as a control intervention. Participants in the control group, if present, were provided with access to a website with links to other websites about childhood brain injury and various brain-injury associations.

The different (online) family problem-solving interventions were investigated in six different studies, resulting in eight published articles. More specifically, one study investigated the CAPS [41–43], two studies examined the TOPS [47,48], one study investigated the non-online FPS [45], one study looked into an online version of the FPS [44] and one examined an adapted version of the FPS [46]. The design method of all of these studies varied (i.e., randomized controlled trials and quasi-experimental pretest/post-test experiments), and outcome measures varied as well (e.g., parent–child conflict is measured in three of the six studies, with two different measures). This makes it difficult to compare the interventions. Overall, the (online) family problem-solving interventions seem to have potential to improve child and family functioning, and therefore the level of activities and participation, of children with (m)TBI. More specifically, the CAPS intervention decreased parent- and teen-reported family conflict and improved everyday functioning in school and in the community of adolescents with complicated mild/moderate TBI. Communication and parent-teen interactions as well as home functioning, behavior, mood or thinking did not change with CAPS [41–43]. The TOPS intervention led to reduced parent-adolescent communication and conflict behavior and decreased parent-reported, but not adolescent-reported, conflict [47,48]. The online FPS intervention seems to be the least effective in improving the level of activities and participation of children with (m)TBI: no effects on parent–child interaction, global parent–child conflict or family functioning were found [45], although improvement was shown for adolescent-reported conflicts regarding school [44].

Several factors influencing effectiveness were identified in the studies investigating the CAPS, the (online) FPS and the TOPS interventions. For one, more improvement in child and adolescent functioning as well as in teen-reported family problem-solving skills and parent- and adolescent-reported child behavior after the (CAPS or adapted online FPS) intervention is related to lower parental education [41,42,46]. This seems to indicate that especially children with mTBI and lower-educated parents can benefit from a problem-solving intervention. Second, in contrast to younger adolescents, older adolescents showed positive behavioral changes and improvements in self-management after the CAPS and online FPS interventions [41,43,46]. Furthermore, the CAPS intervention was especially effective in improving school, work and community functioning, rather than other domains of functioning (e.g., home functioning, behavior and thinking). Last, parent-reported teen internalizing symptoms improved after the TOPS intervention, but only for participants with severe TBI. Taken together, these results indicate that factors such as parental education, age of the child, domain of functioning to be improved and severity of the injury can influence intervention effectiveness.

The effectiveness of the CAPS, the (online) FPS and the TOPS interventions was investigated in groups of children with complicated mild, moderate and severe TBI who were not selected based on their complaints and/or being at risk for these complaints. These interventions should, therefore, be categorized as interventions for the prevention of long-term symptoms. The effectiveness of these interventions in a more selected group of children with TBI remains unknown. Furthermore, since children with complicated mTBI were always analyzed together with children with moderate TBI, it remains unclear what effect these interventions would have on the level of activities and participation and other outcomes in a group solely of children with (complicated) mTBI.

### Cognitive & physical rest

Rest during the acute stage of recovery, reduction of physical and cognitive activities, monitoring symptoms in collaboration with their parents, taking rest breaks after returning to school, spending fewer hours at school, being allowed more time to take exams, having help with schoolwork, gradually returning to sports and reducing time spent with the computer, reading and writing are among the recommendations for managing symptoms after mTBI in children [54–56,59,61]. Cognitive and physical rest recommendation is often part of the care as usual for children with mTBI and is also described in protocols such as the return-to-learn and the return-to-play protocols [62]. However, in reviewing the literature, we encountered only one study investigating the effects of cognitive and physical rest on the level of activities and participation for children with mTBI [31]. To determine if strict cognitive and physical rest was beneficial with regard to postinjury recovery, patients were divided into two groups: one group was recommended to have 1–2 days of rest, while the other group was advised to have strict rest for 5 days. Both groups were recommended to return to activity step by step after the days of rest. Results showed that strict rest caused children with mTBI to report more PCS. Furthermore, in comparison with children who had only 1–2 days of rest, the more rested children experienced a decrease in the level of activities and participation. This is not surprising, since per definition cognitive and physical rest entails restricted level of activities and participation. The effects of cognitive and physical rest on the level of activities and participation over the long term still have to be determined.

## Additional information

### Information & education

Kirkwood *et al*. performed a pilot study investigating a one-time neuropsychological consultation consisting of interviews with parents and children and a standardized battery of tests [57]. Feedback on the results was provided to the families by a neuropsychologist, including general education about concussion, information about injury and noninjury-related factors contributing to the child's specific symptoms and recommendations for addressing any concerns. They found that PCS decreased significantly following the consultation. Unlike the two studies reported above, the study by Kirkwood *et al*. was focused on children with mTBI who were already reporting problems for some time [57]. The finding that the intervention was effective in decreasing PCS in these children is promising, indicating that interventions consisting of information and/or education are suited not only for the prevention of symptoms but also for more specific treatment.

### Follow-up consultancies

In a study by Bell *et al*. follow-up contact by telephone was found to be effective in reducing symptoms after mTBI [63]. This study, however, was performed with children aged 16 years and older and with adults after mTBI and the effect on younger children is unknown (and therefore not in Table 1). Furthermore, the effect on the level of activities and participation was not measured.

### Cognitive & physical rest

From the studies we reviewed for the present article, no consensus can be derived regarding the benefits of cognitive and physical rest for children with mTBI. One study supports rest as an effective form of care after mTBI in children. Independent of when a minimum of 1 week of cognitive and physical rest was described (i.e., 1–7 days, 8–30 days or more than 31 days postinjury), PCS were reduced and cognitive functioning was improved [54]. Another study found no association between the prescription of cognitive rest and the duration of symptoms [53]. While these results seem contradictory, methodological differences between the studies have to be taken into account when interpreting the results. While the first study [54], finding benefits of cognitive and physical rest, examined only the presence of PCS at one time point, the other study [53], failing to find an association between rest and PCS, investigated the duration of symptoms over time. Furthermore, while the first study examined a period of cognitive and physical rest of approximately 1 week, the duration of rest used in the second study is not clear. This should be taken into account, since, as described above, increasing the duration of cognitive and physical rest from 1–2 days to 5 days was found to have negative effects for children with mTBI [31]. The relation between the duration of PCS and the duration of cognitive and physical rest needs further research.

### Combined interventions

Some interventions in children with mTBI are comprised of a combination of components. For example, Gagnon *et al*. used graded guided rehabilitation as their primary intervention [59]. The intervention stops when children are symptom-free. Children who do not remain symptom-free receive a return appointment for re-evaluation, education and a weekly follow-up. This combination continues until the child remains symptom-free. The results of this study suggest that involvement in controlled and closely monitored rehabilitation in the postacute period may promote recovery in children and adolescents who present with slow recovery after mTBI.

Another combined intervention consisted of education and advice on avoiding analgesic overuse, avoiding any opiate medications and encouraging light exercise when PCS persisted for 3 months or longer postinjury [58]. Furthermore, prophylactic medications were selected based on comorbidities by a neurologist with expertise in acquired brain injury and headache disorders. A marked reduction in the frequency of headaches was reported in half of the cases after the intervention, while 45% reported complete resolution of headaches.

A combined collaborative care intervention, consisting of care management, CBT and possible psychopharmacological consultation, was examined by McCarty *et al*. [60]. They found that efforts to systematically implement collaborative care treatment approaches for slow-to-recover adolescents may be useful given the reductions in postconcussive and co-occurring psychological symptoms in addition to improved quality of life.

All of the above-mentioned combined interventions were conducted with children and adolescents who experienced symptoms after mTBI. Although the results are promising, the influence of such interventions on preventing symptoms in the first place was not studied, nor was the influence on activities and participation.

## Evidence from literature on mild forms of brain injury in adults

Interventions designed to reduce symptoms after mTBI in adults have been investigated by several researchers. Providing information with educational brochures or sessions about common symptoms after mTBI, including reassurance of recovery, the likely time course of recovery and information on how to cope with symptoms are among the intervention strategies [64–67], as are neuropsychological assessments and follow-up contact by telephone.

More specifically, for adults with mTBI, Paniak *et al*. [68] showed that an education-oriented single session and a more extensive assessment, education and treatment-as-needed intervention showed similar results on symptom-related, functional and vocational variables 3–4 months after the initial assessment. These results were maintained at the 12-month follow-up, while most improvements in both groups were seen in the first 3 months [65]. Recently similar results were found in a study where a high-risk mTBI group received a doctor's visit in addition to written information, in comparison with a control group receiving only written information [69]. The high-risk group was defined as patients having three or more PCS at 10 days postinjury. The groups did not differ in terms of symptoms, anxiety or depression at the 3-month follow-up. Ponsford *et al*. [66] studied the effectiveness of an extra follow-up moment in which an information booklet on mTBI was given to adults 1 week after visiting the emergency department. The information booklet contained information about mTBI, the possible consequences and time course and coping strategies to deal with these consequences. In comparison with a control group receiving no information booklet, the patients in the intervention reported significantly fewer symptoms and were less stressed at the 3-month follow-up. Nygren-de Boussard *et al*. [70] conducted a systematic review on the evidence of nonsurgical interventions for persistent symptoms after mTBI and also showed the beneficial effects of early, reassuring educational interventions.

Based on the effectiveness of these education interventions, Moulaert *et al*. [71] developed an early neurologically focused intervention for patients with hypoxic brain injury due to a cardiac arrest. Cardiac arrest can lead to hypoxic brain injury which can be comparable to the diffuse damage seen in mTBI. The intervention consists of screening for cognitive and emotional problems, provision of information and support, promotion of self-management strategies and referral to further specialized care if indicated. This intervention was found to be feasible in clinical practice [72] and both clinically effective [73] and cost–effective [74] in comparison with care as usual. Patients in the intervention group had a better quality of life, a better overall emotional state and fewer symptoms of anxiety 1 year postcardiac arrest. Moreover, more people returned to work 3 months postinjury.

Nelson, Sheese and Hammeke propose treatment strategies both on the basis of clinical consensus and the limited evidence base [75]. In addition to education about mTBI, possible persistent symptoms and the natural course of recovery, and reassurance of a good outcome, they suggest reducing activity levels and refraining from hazardous behaviors during the acute phase and a gradual return to lifestyle activities as symptoms permit. Professionals should carefully monitor and offer early intervention for adverse emotional responses, offer symptom-specific treatment when needed, and enable ready access to providers during the first weeks of recovery. Al Sayegh, Sandford and Carson also suggest that information, education and reassurance alone may not be sufficient in reducing PCS [76]. They suggest that cognitive behavioral therapy with psychotherapeutic elements or mindfulness or relaxation techniques may lead to increased improvements. These interventions are directed mainly toward the reduction of PCS and to a lesser extent toward improving the level of activities and participation.

## Conclusion

Most interventions for children with mTBI are designed to reduce symptoms, such as headaches, cognitive problems or other PCS, and are not specifically designed to improve the level of activities and participation. Interventions consisting of information and education seem effective in preventing symptoms when reassurance is combined with information. Reassurance can be optimized by including a follow-up consultation by telephone, during which individual concerns can be addressed. Family problem-solving interventions are effective in improving child and family functioning but have not been investigated for a group of children with only mTBI. There is no consensus on the benefits of cognitive and/or physical rest, but graded activity procedures seem effective in supporting return to school, sports and play. Combined interventions including medication have not been offered as a preventive strategy and the effects of such interventions on the level of activities and participation are unknown.

In addition to studies investigating the effectiveness of interventions for children with mTBI, several protocols and recommendations have been published on returning to activity and returning to school, for which no studies have been made regarding their effectiveness. Protocols on returning to activity can be divided into graded, or step-by-step protocols, and severity-oriented guidelines on how to build levels of activity. The protocols with a graded approach reported the following six steps: no activity; light aerobic exercise; sport-specific exercise; noncontact training drills; full contact practice; and return to play [77–79]. Guidelines that are severity-oriented focus on the severity of symptoms or the numbers of previous concussions. For example, when a child's first concussion is considered to be mild, the guideline would recommend returning to play after being symptom-free for 1 week. For a concussion that is considered severe, the child should be symptom free for a month [80]. Furthermore, severity-oriented guidelines focused on injury-related factors such as confusion, loss of consciousness and post-traumatic amnesia [81,82]. A protocol on returning to school was provided by Master *et al*. [62]. Their step-by-step protocol consisted of the following steps: no activity; gradual reintroduction of cognitive activity; homework at home before schoolwork at school; school re-entry; gradual reintegration into school; and full return to school and cognitive workload. Sady *et al*. recommend a graduated accommodation-based education plan with similar steps [83]. Furthermore, several other recommendations on returning to school can be found in the literature, such as monitoring and support, removal of distractions, excuse or absence from class or activity, and increased time to complete tests and tasks [84]. Unfortunately, most of these protocols focused on sports concussions in school athletes and the effects of these protocols were not examined. Studies on interventions that are set up in order to directly prevent long-term restrictions on participation in activities for children and adolescents after mTBI are, to our knowledge, unknown.

In conclusion, evidence suggests that information and education should always be offered, ideally followed by a consultation in which personalized reassurance is given. The family should be involved and problem-solving interventions seem effective. In addition, clinical recommendations suggest a step-by-step return to cognitive and physical activities, not only restricted to sports.

## Future perspective

This review shows that the literature on early interventions to improve the level of activities and participation in is scarce with regard to pediatric mTBI. There are not many high-quality studies available and the comparability of studies is limited because of variation in population (i.e., separate studies on mTBI), definitions (i.e., the definition of TBI), the aim of the intervention (i.e., prevention or treatment) and outcome domains (i.e., symptoms or activities and participation) and outcome measures. Ideally the first step should be to identify children at risk of long-term problems by conducting longitudinal prospective cohort studies, followed by high-quality randomized controlled trials in which targeted interventions are investigated. Given the current economic pressures in healthcare, these evaluation studies should include analyses of both clinical effectiveness and cost–effectiveness, and consider potential implementation in clinical practice at an early stage. Research studies investigating preventive strategies are challenging because of recruitment (i.e., can we detect all cases), selection bias (i.e., will all cases participate or only those having complaints or fearing consequences) and follow-up (i.e., will all cases remain in the study).

Currently we are conducting a randomized controlled trial in which the early intervention Brains ahead! is being evaluated in terms of effectiveness on participation in activities in comparison with standard care [85]. The Brains ahead! intervention is a combination of screening for mTBI symptoms, psycho-education and follow-up. Outcome is measured 3 and 6 months postinjury. The primary outcome measure is the Child and Adolescent Scale of Participation [86]; in addition, other measures of activities, participation, quality of life and child behavior are performed. We hope to have recruited 140 children by the end of 2017.

**Executive summary**

- Pediatric mild traumatic brain injury may lead to reduced activities and participation in a considerable number of victims.
- Intervention strategies to prevent long-term problems are preferable to treatment of long-term problems.
- Interventions should include information and education on the injury and its possible consequences and include follow-up consultations aimed at reassurance.
- Interventions should be family-centered.
- Step-by-step return to activities is recommended.
- High-quality randomized studies are necessary.
- Consensus on definitions and outcome domains and measurements increases comparability and, therefore, enlarges the evidence base.
